# Novel integrated matching algorithm using a deep learning algorithm for Wi-Fi fingerprint-positioning technique in the indoors-IoT era

**DOI:** 10.7717/peerj-cs.1406

**Published:** 2023-05-31

**Authors:** Safar Maghdid Asaad, Halgurd Sarhang Maghdid

**Affiliations:** 1 Department of Technical Information Systems Engineering, Erbil Technical Engineering College, Erbil Polytechnic University, Erbil, Kurdistan Region, Iraq; 2 Department of Software Engineering, Faculty of Engineering, Koya University, Koya KOY45, Kurdistan Region, Iraq

**Keywords:** Indoors positioning, Indoors localization, RSSI-based fingerprint, OMNeT++, WSN, Machine learning, Deep learning, Augmentation, LSTM

## Abstract

The Internet-of-Things (IoT) has been used with greater frequency to track peoples’ daily activities, particularly those conducted indoors. Wi-Fi technology has been also been used as an alternative to global navigation satellite system (GNSS) technologies to track indoor activities. The received signal strength indicator (RSSI) is widely used to assist in the positioning of Wi-Fi signals. However, the RSSI-based technique suffers from multipath, non-line-of-sight (NLOS) problems and the fluctuation of RSSI measurements via Wi-Fi chipsets. One of the most well-known RSSI-based approaches is to apply the fingerprinting method to do the positioning. However, the fingerprinting-based form has an additional problem due to the lack of RSSI data samples, specifically in harsh area with a huge number of classes or reference points (RPs) and an unstable matching process algorithm. To mitigate the problems of the RSSI-based fingerprinting approach, this research proposes a novel matching process algorithm called Norm_MSATE_LSTM. We first performed the augmentation process to increase the RSSI data records via the Mean Stander deviation Augmentation TEchnique (MSATE). The RSSI records were normalized (norm), and the long short-term memory (LSTM) technique was applied to estimate the correct positions. Finally, the proposed matching algorithm was compared with the stand-alone matching algorithms, including the weighted k-nearest neighbors (WkNN) and LSTM. The results obtained from the experiments and the simulated experiments using OMNeT++ show that the proposed matching algorithm may improve positioning accuracy by 33.1% and 57.5% when only augmentation and augmentation with normalization are applied, respectively.

## Introduction

With the rapid growth and the widespread availability of wireless and Internet-of-Things (IoT) technologies over the past decade, people are paying more attention to location-based services (LBS) in indoor and outdoor environments. Indoor LBS has been widely used for location identification, indoor user tracking, and positioning users’ requests in various IoT environments, including airport terminals, subways and public transportation stations, retail malls, and other indoor settings (Asaad & Maghdid, 2022). Therefore, it is necessary to design effective, precise, reliable, and real-time indoor positioning (IP) systems to meet consumers’ IP requests. The rapid spread of mobile devices and smartphone technology around the world is also driving customer demand for LBS (Liu et al., 2020; Xia et al., 2021).

LBS requirements have been adequately handled and incorporated into the GNSS in outdoor settings. However, the GNSS is unsuitable for IP systems since its signal weakens after passing through interior physical surroundings, resulting in inaccurate location data or entirely blocking GNSS signals (Asaad et al., 2021). IP systems commonly employ Bluetooth (Bai et al., 2020), ultra-wideband (UWB) (Djosic et al., 2021), radio frequency identification (RFID), geomagnetic positioning (Wu et al., 2021), visible light (Guan et al., 2021), ZigBee (Li, 2021), cellular networks (including LTE and 5G) (Chai, Li & Huang, 2020; García, Maier & Philips, 2020), and Wi-Fi (Maghdid et al., 2019) technologies. Each technology has its own set of benefits and drawbacks. Among these, the public availability of Wi-Fi technology at a reasonable cost and with minimal risk to human life makes it the most widely accepted of these technologies. As a result, Wi-Fi positioning has been used indoors as an alternative to GNSS technologies (Asaad & Maghdid, 2022).

In general, wireless positioning techniques, including the time of signal arrival (TOA) (Lim et al., 2021), time difference of signal arrival (TDOA) (Bottigliero et al., 2021; Sadhukhan & Das, 2009), angle of arrival (AOA) (Al-Sadoon et al., 2020), and RSSI fingerprint-based techniques, affect the efficiency of the positioning systems. The aforementioned techniques are based on different measurements, namely the received signal strength indicator (RSSI), and time of flight (TOF). The RSSI measurements can be used to compute the distance between a target device and fixed stations or wireless access points (WAPs) (Maghdid et al., 2019) and to survey the positional environment to estimate the locations of the target device *via* matching algorithms (Zhu et al., 2020). The TOF measurement is also utilized in the TOA, TDOA, and AOA methods to calculate the target device’s distance from the WAPs. However, using these measurements or positioning techniques has its own set of restrictions, making it challenging to complete the procedure accurately.

The RSSI-based fingerprint technique is used widely for indoor positioning due to its simplicity. It does not need to deploy extra hardware and it may be used on most new mobile devices. The RSSI fingerprint-based technique is creating an RSSI fingerprint database for matching or testing sample records during the evaluation and detection process. Distinct RPs in the positioning region obtain different RSSIs of each WAP, which can be utilized as fingerprints. Further, these fingerprints can provide valuable features and unique information in the positioning context (Djosic et al., 2021). Fingerprint-based positioning usually has two main phases: the offline phase and the online phase. The primary task of the offline phase is to create a fingerprint dataset experimentally from obtained RSSI of WAPs at various RPs, which are determined depending on the WAP’s position and the size of the entire positioning area.

Regarding the online phase, the systems are trained *via* machine learning (ML) matching algorithms to identify the incoming online record of RSSI features from WAPs. The most modern and valuable ML techniques for positioning are weighted k-nearest neighbors (Chen, 2021), support vector machine (SVM), random forest (RF), artificial neural network (ANN) (Polak et al., 2021), multi perceptron (MLP), and LSTM as the well-known technique for time series information including Wi-Fi RSSI (Mirdita et al., 2021) and deep neural network (DNN) (Li, Lei & Zhang, 2020). The deep learning (DL) algorithms are more accurate than the rest of the methods. However, the main challenges of the current positioning solutions using the RSSI fingerprint technique are: (1) dynamic indoor structures, which result in dynamic multipath and non-line-of-sight signals; (2) the availability of various IoT devices which are deployed in the vicinity of the same frequency band; (3) the matching algorithms that lack experience with RP prediction; (4) and the solutions that are not deployed in a complex indoors structure with a large enough number of RPs because they are not tested in real scenarios. Lastly, fingerprint-based techniques are time- and effort-consuming, specifically in a wide IoT positioning area, which may lead to a lack of RSSI samples. To overcome these concerns, including the lack of RSSI data samples, simulators can be considered as alternatives to real environments. The common simulators that are used in the positioning include the objective modular network testbed in C++ (OMNeT++) (Irshad et al., 2021), Opnet (Maghdid et al., 2016a), and NS3 (Zhao et al., 2021). In this work, we collected data using OMNeT++, a free simulator that is made available for academic use only. Augmentation procedures may be used to expand the number of RSSI records per each dataset’s accessible RPs (Sinha & Hwang, 2020). Here, an augmentation technique known as the Mean and Standard deviation-based Augmentation Technique (MSATE), is proposed to increase the RSSI samples in each RP.

The contributions of this study may be summarized as follows:

– Building a fingerprinting database by scanning a wide indoors-area with a large number of study halls, laboratories, and offices using an OMNeT++ simulator.
– Expanding the fingerprinting database *via* the augmentation process to increase the performance of the proposed matching algorithms and proving that augmentation can reduce the time and effort needed during the preparation of the fingerprinting database.
– Proposing a novel matching algorithm, namely Norm_MSATE_LSTM. Further, the Norm_MSATE_LSTM algorithm provides better positioning accuracy than stand-alone matching algorithms, including WkNN and LSTM. Additionally, Norm_MSATE_LSTM can offer better positioning accuracy where there is an extensive dataset or a harsh environment, including obstacles and multipath signals.

The remaining sections of this work are organized as follows. The related work section provides a review of the relevant literature. The suggested Wi-Fi fingerprint-based solution is explained in the Proposed Approach section. The experimental environment setting and data collection process are presented in the Experimental Environment and Data Collection section. The proposed solution is compared to many well-known algorithms regarding positioning accuracy and root mean squared error (RMSE) in the Experimental Results and Discussions section. Finally, in the Conclusions section, the achievements and conclusions of this study are summarized.

## Related Work

The LBS applications are used for tracking users (Chen et al., 2020a), routing prediction (Wang et al., 2020), and to predict the revenue from ride-on-demand applications (Guo et al., 2020) in outdoor applications. However, wireless sensing is mainly used for indoor localization (Maghdid et al., 2019) and event detection (Sharma & Singh, 2021) in indoor applications. Due to its availability in crowded areas and its low cost, Wi-Fi is the most commonly used technology in IP systems. Specifically, the Wi-Fi RSSI-based fingerprinting technique predicts the users’ locations *via* existing WAPs signals in the area. However, this technique has many limitations, from the dynamic multipath to the inaccurate matching process. Therefore, this section presents and investigates the limitations of the current IP solutions where the RSSI-based fingerprinting technique is used.

The current fingerprint-based solutions use a modified version of the kNN which integrates the weighted Euclidean distance metric based on the attenuation law of spatial WAPs-RSSI values. The recommended weighted-kNN technique can determine the user’s position. Although this solution reduces positioning errors, it takes a long time to predict users’ positions due to computational requirements. Therefore, the researchers in Lan & Li (2019) integrated a clustering method with kNN to improve the similarity values in fingerprint features. This method also provides an efficient IP solution that addresses the computational complexity problem of the kNN classifier, which grows linearly with the number of observations, and enhances the positioning accuracy according to experimental data. However, the experimental field was small, with just five WAPs and 120 RPs to choose from, and is insufficient for larger needs of university campuses and airports, where there will be more RPs. Furthermore, clustering approaches can improve positioning efficiency by lowering storage and search overheads in fingerprinting-based systems (Sadhukhan et al., 2021).

Song et al. (2019) developed Wi-Fi fingerprinting and CNN-based IP solutions for multi-building and multi-floor positioning. Their proposed solution combines a stacked auto-encoder (SAE) and one-dimensional CNN methods. The SAE was applied to extract essential features from the sparse RSSI data of WAPs, whereas the CNN was used to perform the classification process. The authors used simulations with two different datasets (Torres-Sospedra et al., 2014; Lohan et al., 2017) as well as their in-house dataset, UTSIndoorLoc, to validate the solution. The solution substantially improved the positioning performance on the newly UTSIndoorLoc dataset, with a floor classification success rate of 94.57% and a mean positioning error of 7.60 m. Nonetheless, the positioning error rate of more than 3 meters restricts its potential for real-world deployments.

Chen et al. (2020b) proposed a deep LSTM solution based on local features that utilize the RSSI values to predict the users’ position. In a 55 x 50 m office, an average positioning error of 1.75 m was attained, with a maximum error of roughly 10 m. Although it is clear that the method improves performance, outliers may still occur due to the rise and fall of the RSSI values. Furthermore, as the network deepens and the site survey increases, the number of RPs will noticeably increase, and the solution will cease to function correctly. In situations with a substantial number of RPs, DL approaches will not perform well, mainly when there are a small number of RSSI samples per RPs, which leads to the failure of the DL learning process. There are two possibilities for solving these concerns. The first is to spend more time and energy in the physical surveying environment to gather additional RSSI recordings. The second is based on augmentation techniques, which include oversampling the RSSI data records using the previously gathered dataset (Sinha & Hwang, 2020).

The location of the anchor nodes deployed in the positioning contexts in fingerprinting-based solutions is one of the most critical factors affecting the accuracy of the positioning systems. Sadhukhan, Dahal & Pervez (2017) examined the influence of the beacon node location on the results of numerous clustering-based approaches to minimize the localization time. They suggested an ideal beacon node arrangement approach that provided coverage visibility inside the positioning environment to enhance IP accuracy in all of the evaluated clustering approaches by measuring positioning time and error as the two performance indicators.

In Roy et al. (2021), a solution for dealing with the uncertainty in RSSI Wi-Fi fingerprint-based localization performance was presented. To handle various smartphone settings within the crowdsourcing era, the authors utilized the Dempster–Shafer belief theory to determine the weights of several classifiers, including SVM, KNN, Bayes network, and K*. K* is an instance-based and rule-based learning-optimized lazy family classifier. To increase the classifiers’ accuracy, the means and the standard deviations were included as new features, along with RSSI records. They demonstrated that weighted ensemble techniques outperformed traditional ensemble techniques in IP systems. However, due to its lack of resilience and increased complexity, the solution’s chance of being implemented in the actual world is slim.

It is clear from the literature that most of the fingerprinting-based techniques have been based on RSSI measurements. However, fingerprinting can be based on other features extracted from channel state information (CSI) in the acquired traffic packets. Zhu et al. (2021) utilized CSI as the target device fingerprint to extract features and a broad learning system (BLS) training design. They employed the Bayesian classifier to investigate the effectiveness of the proposed localization solution. The authors aimed to reduce the training time compared to the state-of-the-art solutions.

Moreover, several studies on self-locating sensors in indoor IoT contexts have developed recently. The authors of Garcia et al. (2009) propose a method for wireless sensors to find themselves in interior environments. Their method is based on a WLAN RSSI readout at the building level. The technique involves a training phase and a heuristic algorithm that employs fingerprinting to identify sensor positions. Another breakthrough in Garcia et al. (2007) was that wireless sensors could determine their self-location using RSSI from WLAN technology. The authors conducted their experiments indoors with walls and other obstacles, multiple interfering sources, multipath impacts, and humidity and temperature fluctuations. The sensor’s self-location was determined using neuronal networks based on the RSSI dataset from the training phase. However, the attained positioning average error with the neural network-based approach was approximately 2.5 m and still requires improvement.

A deep learning neural networks (DNN)-based solution was presented in Labinghisa & Lee (2021). This solution was built on the notion of virtual Wi-Fi access points (VWAPs) to increase the quantity of WAPs without adding extra hardware. In a limited testing environment, the experiment utilized 20 RPs from the existing three WAPs and two VWAPs. The additional WAPs made it easier to gather more mobile users’ device RSSI values for DNN training. However, the ability to reach users successfully in an oversized and complex positioning environment is not guaranteed.

It is clear from the literature review that the majority of indoor positioning solutions based on the RSSI fingerprinting technique suffer from low positioning accuracy or the positioning solutions are tested in limited or unrealistic areas. Therefore, the current RSSI fingerprinting techniques are not ideal for indoor localization. This work proposes an algorithm to provide better positioning accuracy, tested in a wider area, in comparison with most of the state-of-the-art (SOTA) works that have been reported in the literature to date.

## Proposed approach

**Figure 1:**
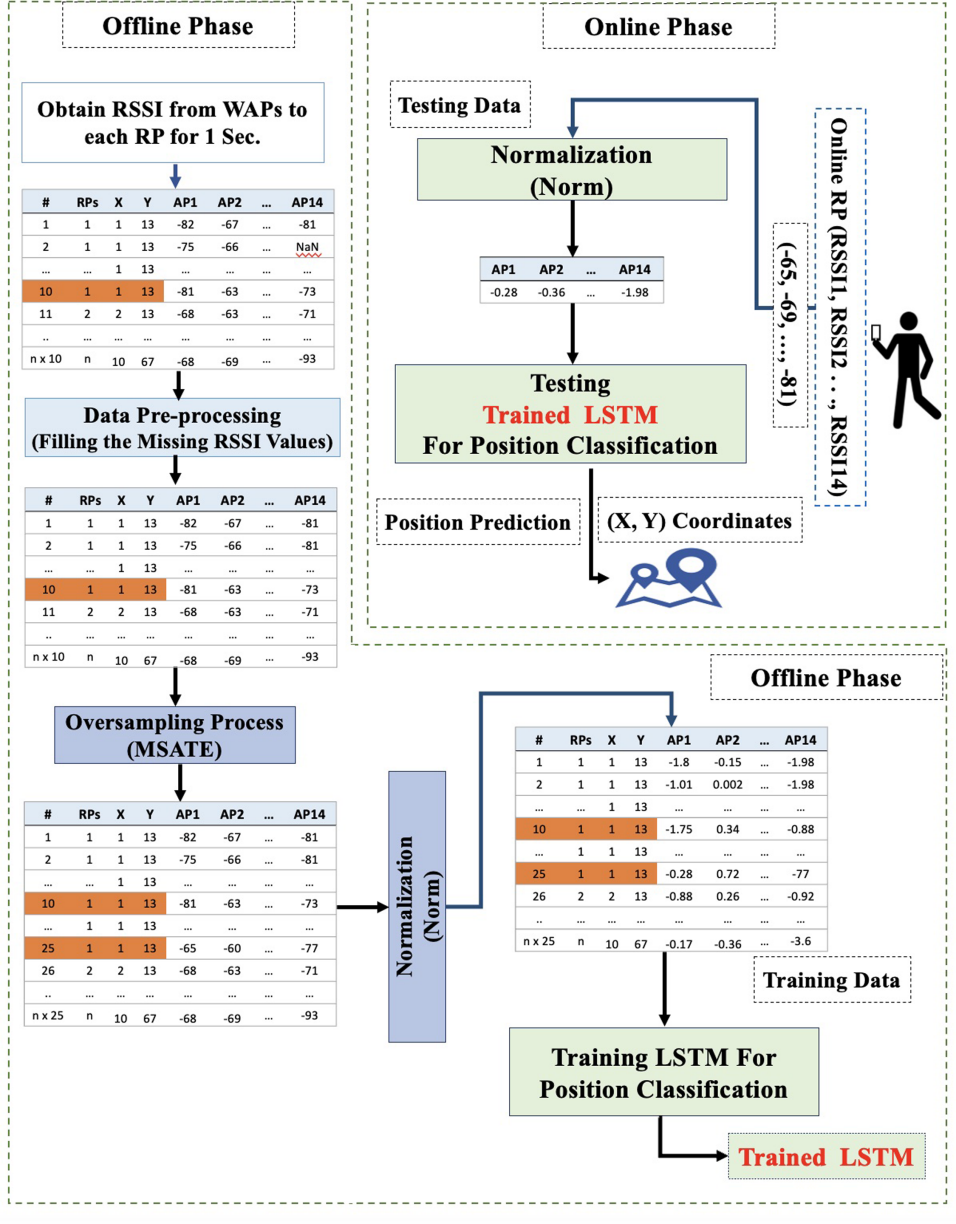
Proposed Wi-Fi fingerprint-based solution diagram (norm_MSATE_LSTM).

This section explains all of the proposed Wi-Fi fingerprint-based IP solution steps, as shown in Fig. 1.

### Methodology

The first step of the proposed approach is collecting an extensive dataset of WPA RSSI in the offline phase. However, as explained in the next section, a preprocessing technique should be applied to construct a comprehensive dataset in a harsh environment, including RSSI multipath issues. Further, a Mean and Standard deviation based Augmentation TEchnique (MSATE) was proposed to enhance the quantity of RSSI samples for each class or RP. One of the methods used during data analysis to increase the quantity of RSSI data samples is known as data augmentation. Data augmentation involving the creation of freshly-produced synthetic RSSI data based on previously gathered datasets, or the addition of slightly modified copies of exisiting RSSI data, was used for data analysis. This serves as a regularizer and aids in reducing overfitting while training a DL model. However, in addition to the benefits, augmentation has certain limitations. The fundamental disadvantage of data augmentation is data bias, which means that the enhanced data distribution may deviate significantly from the original. Because of this data bias, conventional data augmentation strategies perform sub-optimally for time series data (Wen et al., 2021; Wong et al., 2016). Therefore, to determine the best data augmentation approach, additional research is required to generate new or synthetic data records with advanced applicability. The suggested approach, MSATE, produces a new N number of RSSI values for each WAP at a specified position (p) based on the mean and standard deviation computation for the pre-collected RSSI values at point (p). There will therefore be N new RSSI records at (p). The augmented records are expressed in Eq. (1):

(1) \begin{eqnarray*}re{c}_{j,p~Aug}=(RS{S}_{1,p~Aug},RS{S}_{2,p~Aug},RS{S}_{3,p~Aug},\ldots ..RS{S}_{M,p~Aug})\end{eqnarray*}

where *rec*_*j*,*p* *Aug*_ is the jth augmented record at point p (for this study j is between 1 ≤ j ≤ 15), M is the number of the utilized WAPs (for this study M is equal to 14 WAPs), and *RSS*_*M*,*p* *Aug*_ is the augmented RSS values for each WAPs at point p, as it is expressed in Eq. (2):

(2) \begin{eqnarray*}RS{S}_{i,Aug}=RS{S}_{m,i,p}\pm RS{S}_{std,i,\ast }+Rand\end{eqnarray*}

where the i value is between (1 ≤ i ≤ 14) since the number of the utilized WAPs is 14, *RSS*_*m*,*i*,*p*_ is the mean of ith WAPs RSS values at point p, *RSS*_*std*,*i*,∗_ is the mean of the standard deviations (*RSS*_*std*,*i*,*p*_) of all points for each WAPs, as presented in Eq. (3), and Rand is the randomized value between (−2 ≤ Rand ≤ +2).

**Figure 2:**
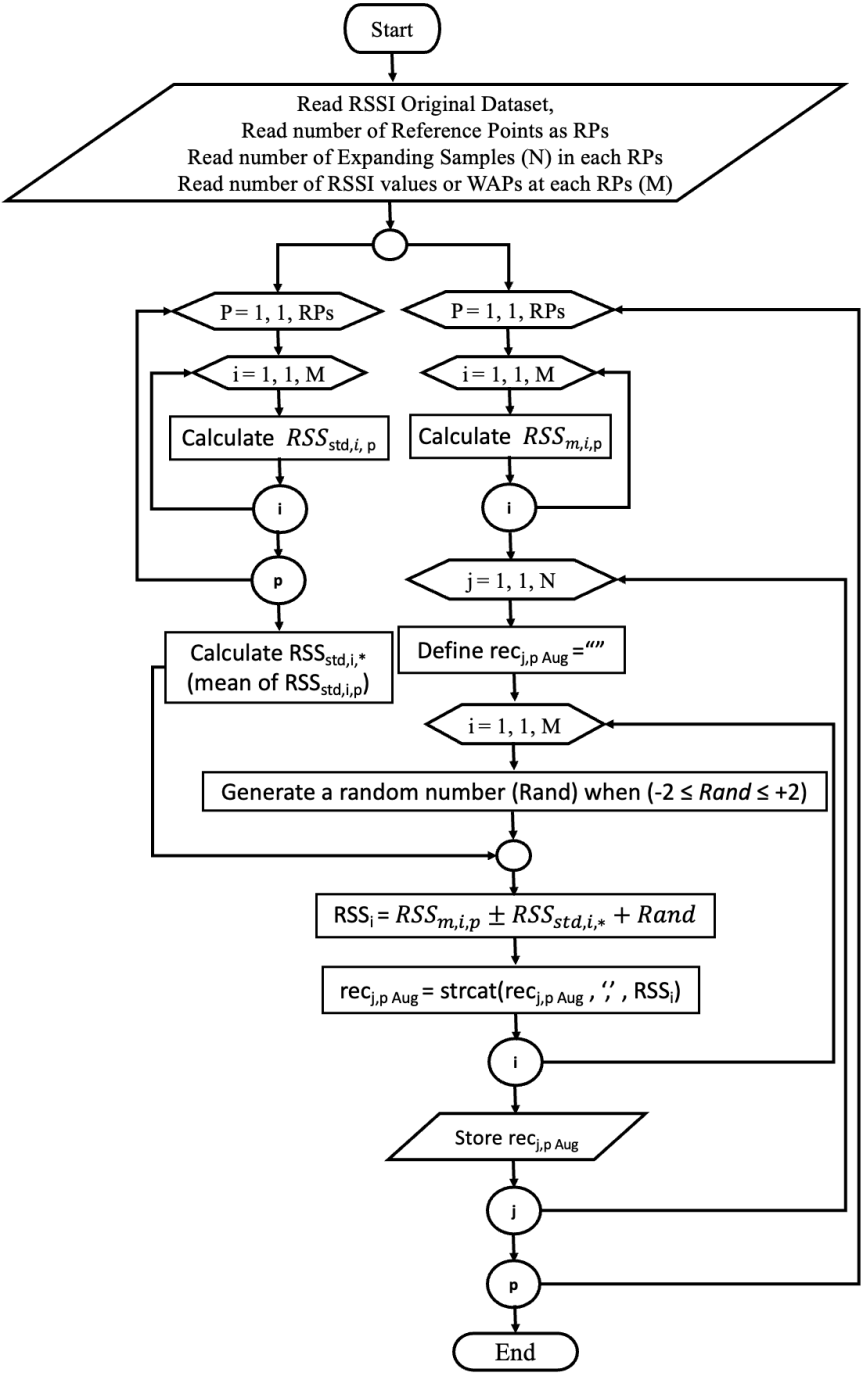
Proposed augmentation technique (MSATE) flowchart.

(3) \begin{eqnarray*}RS{S}_{std,i,\ast }= \frac{\sum _{p=1}^{no\text{_}RPs}RS{S}_{std,i,p}}{no\text{_}RPs} \end{eqnarray*}

where no_RPs is the number of RPs of the setting area, and *RSS*_*std*,*i*,*p*_ is the standard deviations of the RSS values of ith WAP at point p. The proposed augmentation procedure is depicted in the flowchart below, Fig. 2.

In the second step, the proposed solution applies the normalization process over the expanded dataset. Normalization is a method that is frequently used as a preprocessing mechanism in the preparation of machine learning data. The purpose of normalization is to maintain the RSSI values’ ranges while converting the values of a dataset’s numeric RSSI columns to a comparable scale. Not every dataset has to be normalized for machine learning and it is only necessary when features have separate ranges. Here, the normalization was done to reduce fluctuation problems, particularly those that arise when using a variety of platforms and devices to improve the performance of the LSTM (Maghdid et al., 2022). The RSSI values were then transformed to a comparable scale that was used to assist in LSTM training. The z-score method was used as the applied normalization method, which generates a data set with a mean of 0 and a standard deviation (std) of 1. This scaling approach works well when the data follows a Gaussian distribution (normal distribution) (Tabbakha et al., 2021). A snapshot of the normalization formula is expressed in Eq. (4).

(4) \begin{eqnarray*}re{c}_{j~Norm}=(RS{S}_{1~Norm},RS{S}_{2~Norm},RS{S}_{3~Norm},\ldots ..RS{S}_{M~Norm})\end{eqnarray*}

where *rec*_*j* *Norm*_ is the jth record in the dataset, M is the number of the utilized WAPs (for this study M is equal to 14 WAPs), and *RSS*_*M* *Norm*_ is the normalized RSS values for each WAPs at any records, as it is expressed in Eq. (5):

(5) \begin{eqnarray*}RS{S}_{M,~Norm}= \frac{RS{S}_{M}-RS{S}_{m,i}}{RS{S}_{std,i}} \end{eqnarray*}

where the i value is between (1 ≤ i ≤ 14) since the number of the utilized WAPs is 14, *RSS*_*m*,*i*_ is the mean of ith WAPs RSS values, and *RSS*_*std*,*i*_ is the standard deviation of the RSS values of ith WAP.

**Figure 3:**
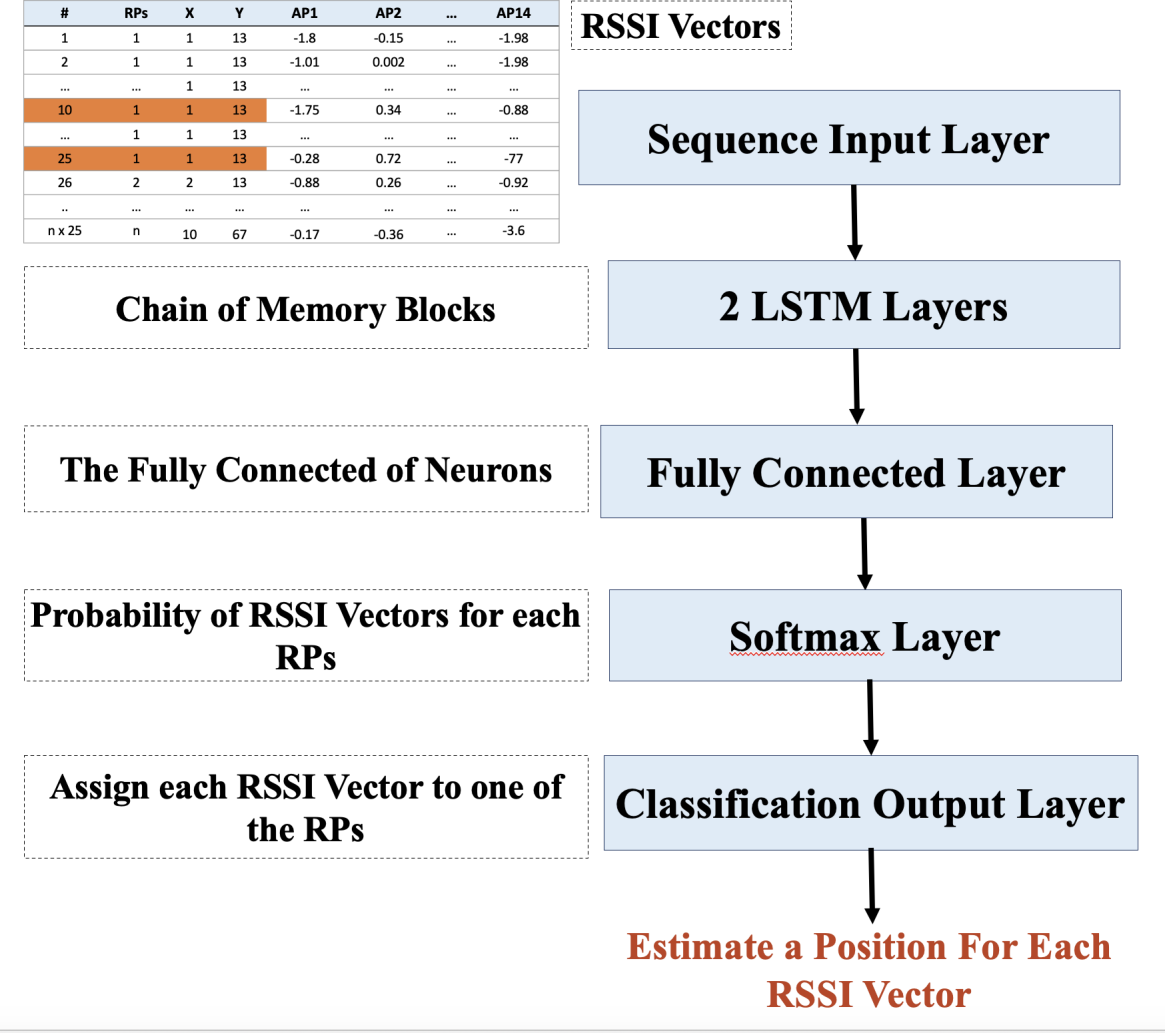
Design of the LSTM.

The third step uses the LSTM as a matching approach. It is used for position estimation after the RSSI data preparation. As a rule of thumb, the LSTM network is an extension of RNN utilized in deep learning and it can effectively train enormous structures of data. However, using LSTM without applying data normalization and augmentation will not provide accurate results, specifically for positioning estimation; this is due to the large number of RPs available. To this end, the LSTM technique was used after making some data preparations, including RSSI data augmentation (MSATE) and normalization (Norm). The data preparation addressed the challenge of too many RPs/classes that were available throughout the setting area. However, the aim of using the LSTM was to minimize the time and memory complexity of the WkNN. Figure 3 depicts the design of the LSTM and shows that the network will be fed the sequence from the input layer, which is a series of normalized RSSI values for each RP or fingerprint.

Afterward, the LSTM layer was connected to the fully-connected layer. The fully-connected layer was in charge of connecting its nodes to all other nodes in the LSTM layer. This layer is responsible for deep data feature analysis, with hidden layers automatically performing feature extraction. Each node in the fully connected layer can be used as an activation function across the RSSI vector on a linear combination of all the features learned by the previous layers. A set of weights were used as the parameters for the linear combination expression. These weights were applied to each layer (Abdul & Al-Talabani, 2022). The last fully-connected layer divided the node outputs into as many nodes as there were classes in the classification task, which, in this case, was 2,214 classes. As a result, in the last fully-connected layer, the number of classes equaled the output size parameter. Because the region included several classes/RPs, the Softmax layer was utilized to compute each class’s probability using the classification layer’s network inputs.

#### LSTM background

**Figure 4:**
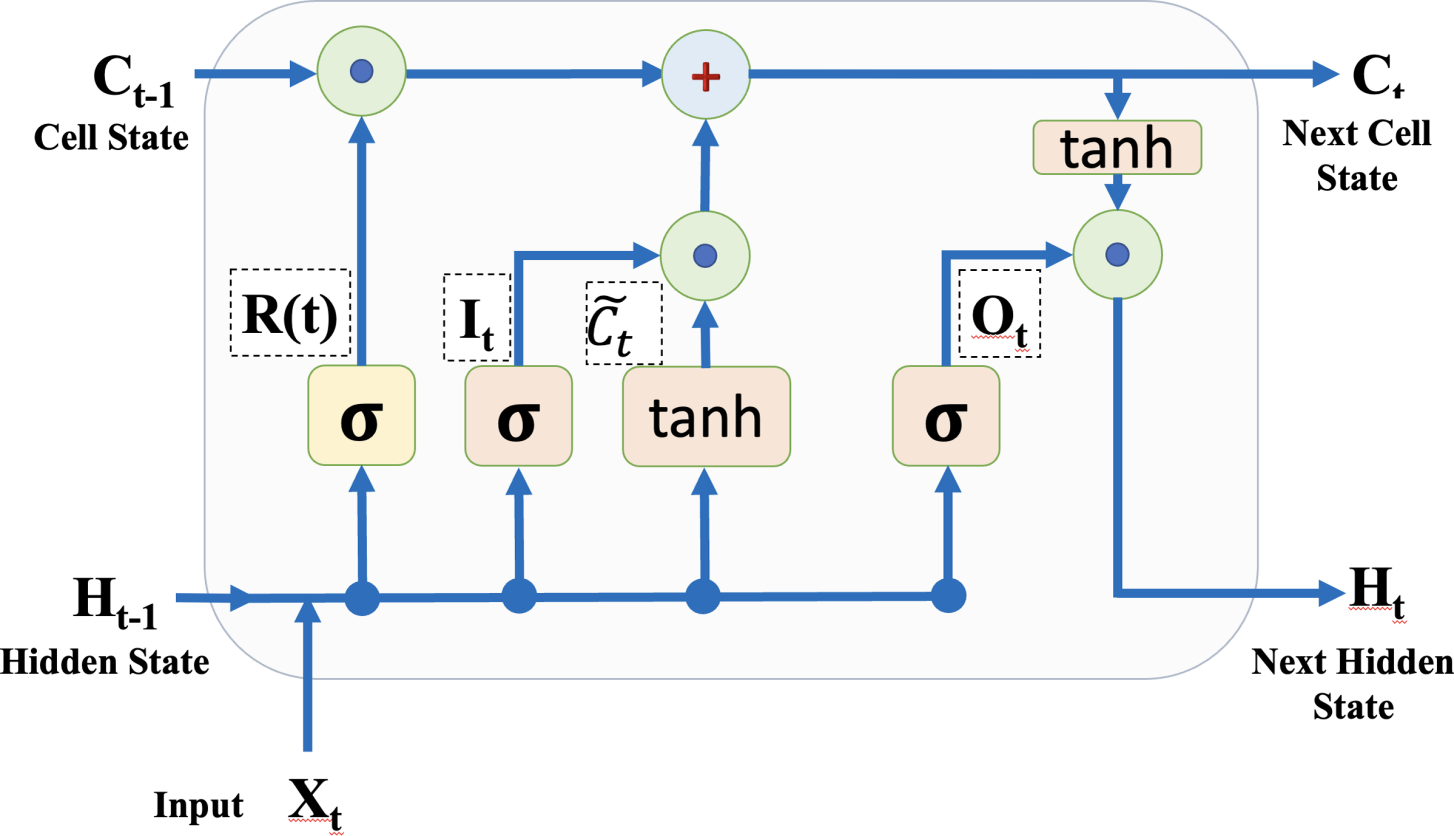
A memory block of LSTM layer.

The LSTM network is an extension of RNN utilized in deep learning. In the network structure, the LSTM comprises a more complex repeating module. The primary distinction between regular RNN and LSTM is that the LSTM can detect long-term dependencies. It employs an architecture that can overcome the gradient vanishing problem. The LSTM is a deep learning algorithm that classifies and regresses time-series data like sounds and RSSI values by taking feature changes into account at each time step. The LSTM layers establish long-range connections between the sequence data’s RSSI values. The structure of a memory block LSTM is depicted in Fig. 4 to clarify the LSTM layer. Equations (6) and (7) calculate the cell and hidden states, respectively (Abdul, Al-Talabani & Ramadan, 2020).

(6) \begin{eqnarray*}{C}_{t}=R \left( t \right) \odot {C}_{t-1}+{I}_{t}\odot \widetilde {{C}_{t}}\end{eqnarray*}

(7) \begin{eqnarray*}{H}_{t}={O}_{t}\odot \tanh \nolimits \left( {C}_{t} \right) \end{eqnarray*}

where ⊙ denotes the vectors’ element-by-element multiplication and tanh() is the hyperbolic tangent function, which is the state activation function. The input gate and forget (remember) gate are denoted by *I*_*t*_ and $R \left( t \right) $. The cell candidate gate is presented by *C*_*t*_, the candidate for cell state is indicated by $\widetilde {{C}_{t}}$, which is determined through Eq. (8), and the output gate is *O*_*t*_ in this study.

(8) \begin{eqnarray*}\widetilde {{C}_{t}}=\tanh \nolimits ~({W}_{C} \left[ {H}_{t-1},{X}_{t} \right] +{B}_{c})\end{eqnarray*}

where $\widetilde {{C}_{t}}$ indicates the weight for the input gate, *X*_*t*_ is the input at timestamp t and *B*_*c*_

is a bias for the input gate.

## Experimental environment and data collection

This section presents the experimental environment setting and data collection process. This is followed by the data preprocessing stage. Finally, the proposed Norm_MSATE_LSTM classifier’s configuration and operating concept are described.

### Environment setting

**Figure 5:**
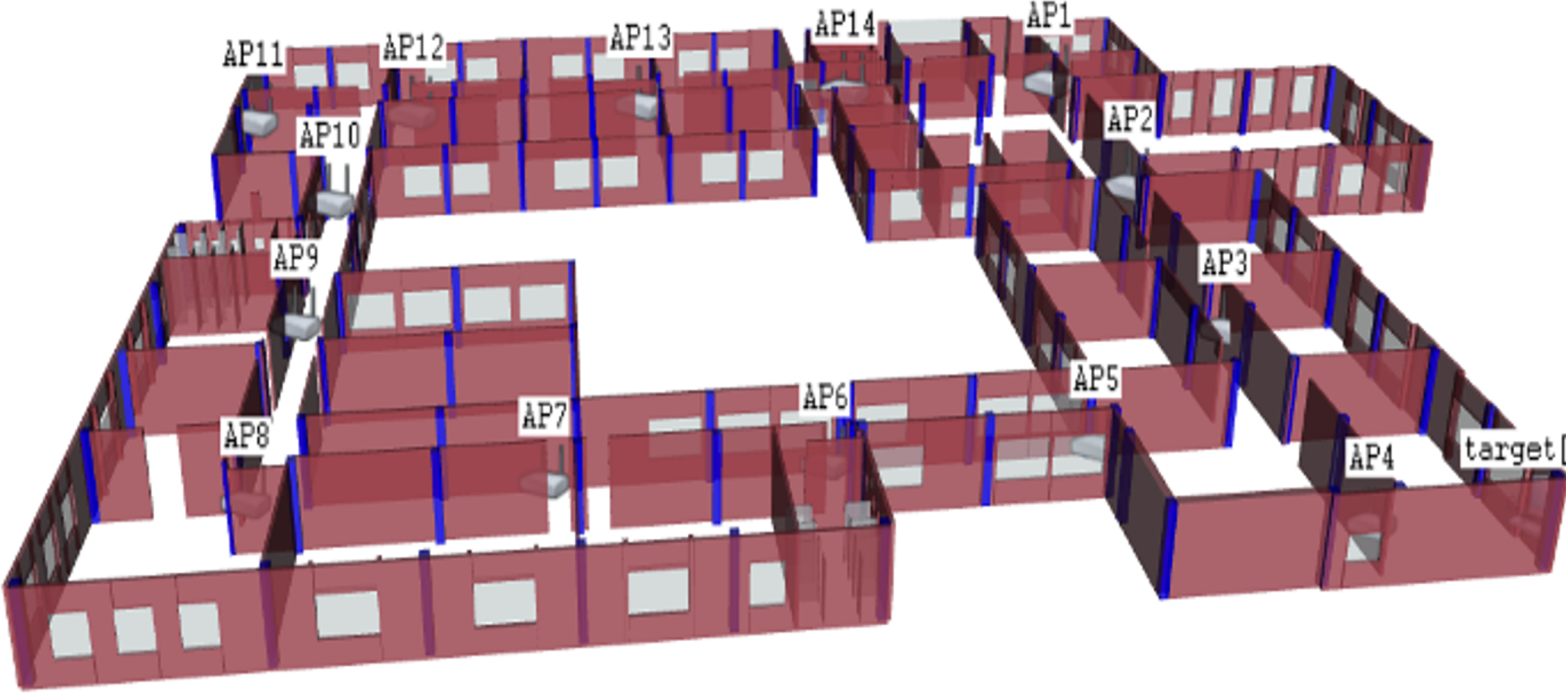
The OMNeT++ based environmental area; third floor of the Faculty of Engineering from Koya University.

The OMNeT++ simulator simulated the third floor of the Faculty of Engineering building from Koya University. This floor was chosen and constructed as an experimental setting in the proposed solution. The simulated area was approximately 56 ×77.6 square meters, and was comprised of four corridors, sixteen study halls, four computer laboratories, nine staff offices, three restrooms, and a large 13m ×19 m meeting hall. A total of 14 WAPs were deployed in the survey area, as depicted in Fig. 5.

### Data collection

**Figure 6:**
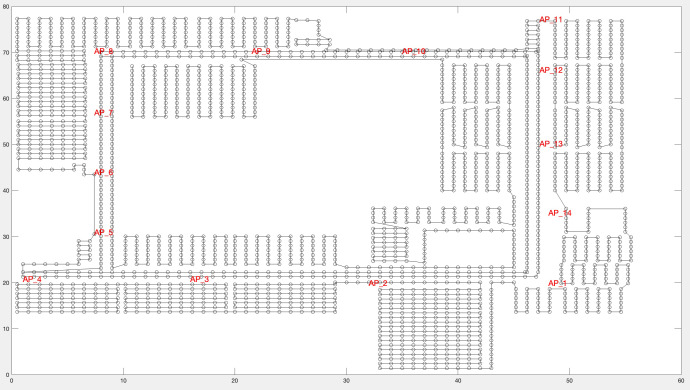
Region division of many RPs.

The entire region of the third floor was included for data collection. The area was divided into grids of 1 m ×1 m, dividing the region into 2,214 points, as illustrated in Fig. 6. At each sample point, RSSI data was recorded for one second. A total of 22,140 records were collected.

A set of parameters were configured for the overall WAPs and a smartphone was simulated in the network to deploy the simulation. Table 1 shows the primary network parameters used to construct the experimental environment in the OMNeT++ simulator, with a simulation period of 2,214 s.

**Table 1:** Environmental settings and variables.

Deployment area	56 m 77.6 m
Number of WAPs	14
Number of target device	1
Simulation duration	2214s
Carrier frequency	2.4 GHz
Bitrate	54 Mbps
Wlan type	Ieee80211MgmtAp and Ieee80211MgmtSta
Number of channels	14
Scan type	Passive
Mobility model	Modified bonn motion mobility
Transmission power	2W
Packet interval (beacon Interval)	100 ms
Path loss model	Rayleigh fading
Propagation model	Constant speed propagation
Obstacle loss type	Dielectric obstacle loss

### Data preprocessing

Due to the restricted coverage range of the Wi-Fi, neighboring interfering devices, the existence of obstacles, and other factors, not all WAPs were detected among all of the RPs. The missing WAP RSSI values were required to have an RSSI value before data processing could take place. As a result, the missing RSSI values were fed with pre-known RSSI data from the related WAPs and at the same RPs. Additionally, the RSSI value dataset ranged from -17 dBm to -100 dBm and records with less than -100 dBm, or greater than -17 dBm, were ignored. After this process, ten RSSI records were kept at each RP. Consequently, there were 2,214 RPs and 22,140 preprocessed RSSI record RPs in the entire region.

### Setting up the parameters of the LSTM and WkNN

The ML approach, LSTM, was used to train the model based on RSSI data obtained from the OMNeT++ environment and augmentation process. The trained approach was configured and tuned to provide improved positioning results.

The selection of hyperparameters was given special consideration here. The LSTM model was updated until the optimal hyperparameters were chosen and an optimized LSTM model was obtained. The Bayesian optimizer was then applied to determine the optimal hyperparameters and training choices for the LSTM model. The Bayesian optimizer is a practical approach to sweep hyperparameters in such experiments. In this research, the MatLab toolbox’s Experiment Manager was used to look for a combination of hyperparameters to improve the accuracy of the proposed solution after giving a range of values for each hyperparameter. Table 2 shows the set of parameters used to train LSTM.

The RSSI dataset was divided into two portions during the experimental setup to evaluate the proposed solution, including the LSTM and WkNN. The training used 80 percent of the whole fingerprint data, including the original and augmented data. Twenty percent of the remaining data was utilized during the testing phase. As indicated earlier, each RP had 25 RSSI recordings after preprocessing and augmentation. During data separation (80% for training, 20% for testing), every fifth record out of every five records was chosen for testing purposes, and the remaining four records were used for training.

The proposed Norm_MSATE_LSTMT solution was compared with the WkNN technique to evaluate its performance. When estimating the position (x, y) of a sample RSSI vector, we used the WkNN at a city block distance (Prabhakar & Rajaguru, 2016). An exhaustive search algorithm was used as the nearest neighbor search method. In this work, the value of k was chosen empirically and was initialized at 3. The weight values (w1, w2, w3) were added for the three selected RP coordinates {(x1, y1),(x2, y2),(x3, y3)}. The weight values were updated for each iteration when applying the WkNN algorithm, as expressed in Eq. (9).

(9) \begin{eqnarray*} \frac{S=\sum _{i=1}^{k} \frac{1}{{d}_{i}} }{{w}_{i}= \frac{ \frac{1}{{d}_{i}} }{S} } \end{eqnarray*}

where *d*_*i*_ is the distance between target RSSI sample vector at (x, y) coordinates and ith RSSI vector with (xi, yi) coordinates from the three selected RPs. The target coordinate (x, y) was then calculated by weighting the coordinate positions using Eq. (10).

(10) \begin{eqnarray*} \left\{ \begin{array}{@{}l@{}} \displaystyle x={\mathop{\sum \nolimits }\nolimits }_{i=1}^{3}{w}_{i}{x}_{i} \\ \displaystyle y={\mathop{\sum \nolimits }\nolimits }_{i=1}^{3}{w}_{i}{y}_{i} \end{array}. \right. \end{eqnarray*}

**Table 2:** Approved set of parameters.

Parameters	Value	Note
LSTM layers	2 layers with 300 hidden units	
dropoutLayer	0.6	
Number of classes	2,214	2,214 RPs
Input size	14	14 WAPs
Optimizer	Adam	
Validation frequency	691	
MiniBatchSize	64	
Number of epochs	140	
Initial learning rate	0.001	

## Experimental results and discussions

**Figure 7:**
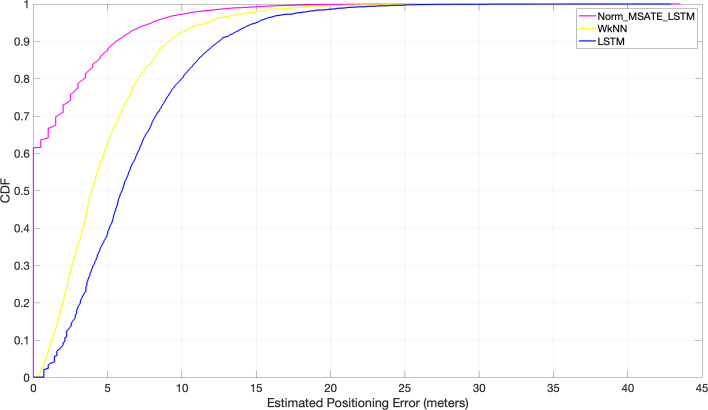
Comparison of cumulative positioning error probability (Norm_MSATE_LSTM *vs* WkNN *vs* LSTM).

A set of experiments were conducted to assess the proposed approach’s performance in a simulated setting. The results were compared to the two cutting-edge matching algorithms (WkNN Zhou, Yang & Chen, 2021 and LSTM Maghdid et al., 2022). The experiment was conducted on the RPs throughout the entire region. After feeding each of the aforementioned algorithms, WkNN and LSTM, and the proposed Norm_MSATE_LSTM, the obtained RMSEs were 4.8 m, 6.8 m, and sane as genuine meters, respectively, presented in Table 3. Further, the proposed method identified almost 62% of the positions correctly, *i.e.,* the 62% positioning error was zero. While, with the WkNN alone, 62% of the positioning error was approximately 5 m, and it was more inaccurate with LSTM, with a 62% positioning error of about 7.2 m as illustrated in Fig. 7.

**Table 3:** Comparison of localization accuracy and RMSE.

Methods	WKNN	LSTM
Original Dataset	4.8008 meter	6.7990 meter
Normalization	4.4049 meter	4.6351 meter
MSATE	1.9315 meter	4.3071 meter
Norm_MSATE	1.9374 meter	1.6797 meter
(Sinha & Hwang, 2020) (Augmentation)	4.2216	6.4417
Norm_(Sinha & Hwang, 2020)	4.2307	3.9509

**Figure 8:**
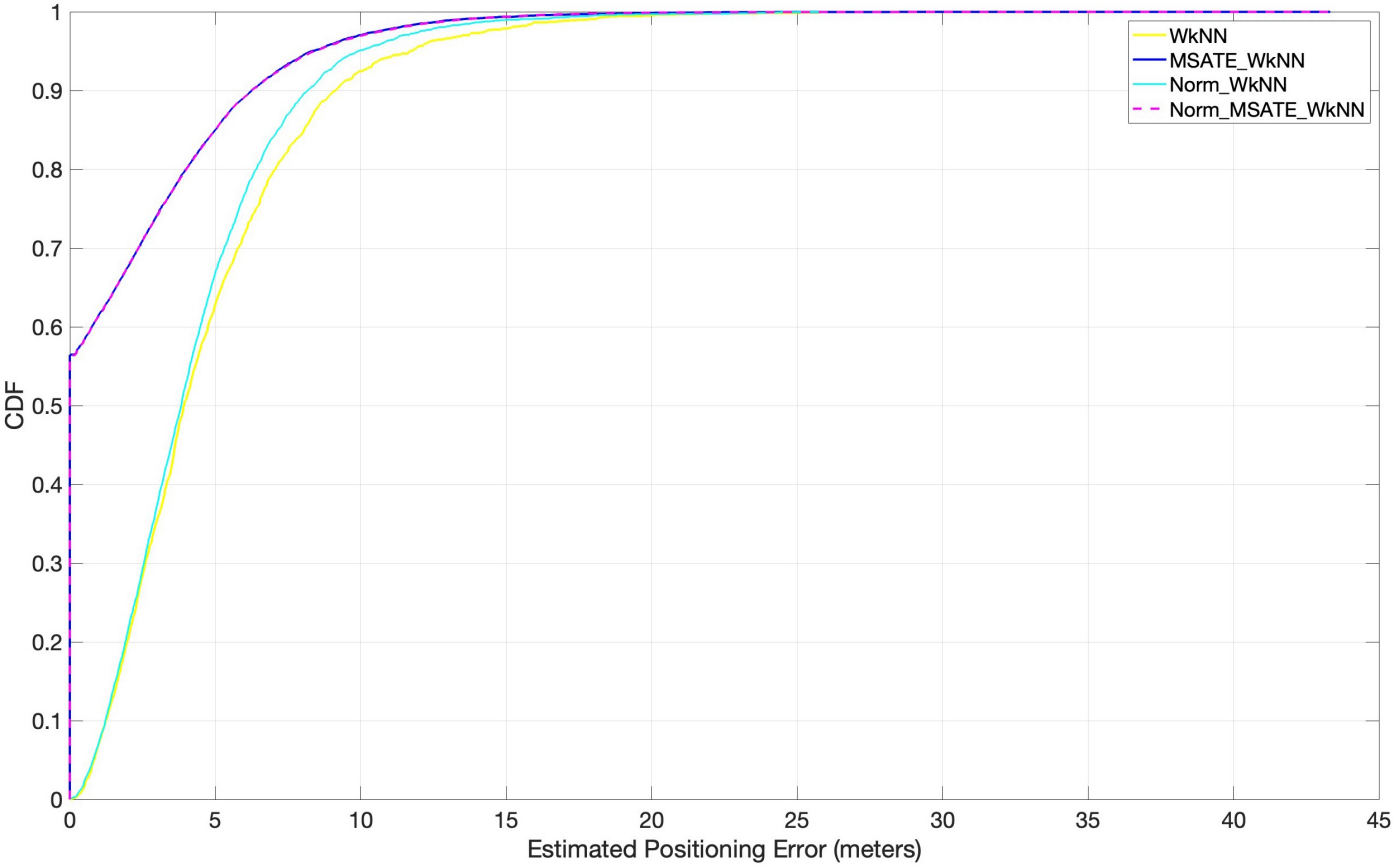
Comparison of cumulative positioning error probability (effectiveness of MSATE with WkNN).

Several experiments, as depicted in Fig. 8, were conducted to evaluate the impact of the proposed augmentation technique on similarity-based machine learning algorithms in indoor positioning (*i.e.,* indoor positioning accuracy). Positioning accuracy is defined as the cumulative percentage of positioning error within a certain distance. WkNN was implemented in accordance with the following conditions:

(1) Norm_WkNN, in which the WkNN was fed with the original normalized dataset without considering the augmented dataset records.

(2) MSATE_WkNN, *i.e.,* merely applying the proposed augmentation process over the original dataset to increase the number of samples. The WkNN was fed with all the dataset records without applying normalization over the data.

(3) Norm_MSATE_WkNN, in which the WkNN was fed with data after applying both the augmenting and the normalizing on the original dataset. Figure 8 shows the experimental findings.

It is evident from Fig. 8 that the proposed augmentation technique, MSATE, with the WkNN (MSATE_WkNN) was more successful than WkNN alone in terms of positioning accuracy improvement. MSATE_WkNN was capable of identifying approximately 57% of the testing records correctly. This was due to the effectiveness of the proposed augmentation technique. The findings of the Norm_WkNN experiment indicated that normalization alone did not enhance the WkNN much when the WkNN was used to predict the locations based on the originally-collected data after normalization. This was due to role of the similarity process in that method. Due to the ineffectiveness of the normalization, Norm_MSATE_WkNN and MSATE_WkNN provided identical results, as seen by the final results.

**Figure 9:**
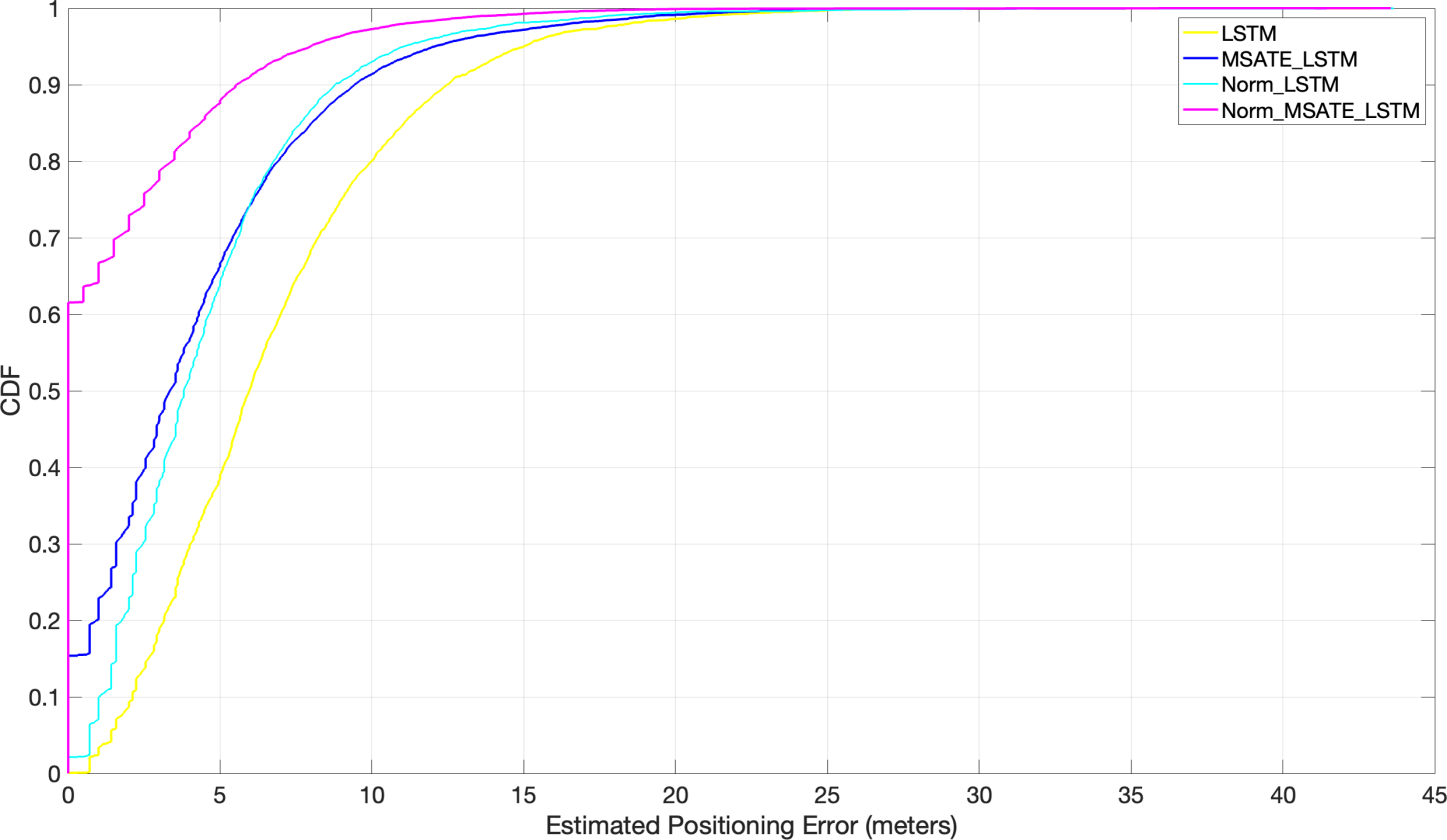
Comparison of cumulative positioning error probability (effectiveness of MSATE with LSTM).

In addition, the efficiency of the suggested augmentation strategy with the DL techniques was evaluated in a variety of trials under varying situations, as shown in Fig. 9. It is evident from the figure that the normalization and augmentation processes on the original dataset contributed little to the positioning improvement with LSTM in both Norm_LSTM and MSATE_LSTM scenarios. In contrast, when both the augmentation and normalization operations were carried out on the original dataset using the LSTM (the suggested method, Norm_MSATE_LSTM), the positioning accuracy was significantly enhanced. According to the figure, the likelihood of accurately recognizing the testing records was 62%, 17%, 2%, and 0% for the Norm_MSATE_LSTM, MSATE_LSTM, Norm_LSTM, and LSTM scenarios, respectively.

In order to realize the effectiveness of our proposed augmentation technique (MSATE) *vs.* the techniques proposed in the literature, the MSATE was compared with Sinha & Hwang (2020). Sinha & Hwang (2020), proposed an augmentation technique to oversample the dataset samples precisely when there was a lack of RSSI samples. This created new RSSI samples that mimicked the original RSSI readings regarding each WAPs; the newly generated RSSI samples were then used to expand the training dataset. In their study, only RSSI values from each RP were used to augment. The RSSI value at each RP was chosen randomly and entered into a new CSV file, increasing the amount of the data compared to the original dataset. The resilience comes from the fact that the enhanced data pattern closely resembled the RSSI data samples prior to augmentation.

**Figure 10:**
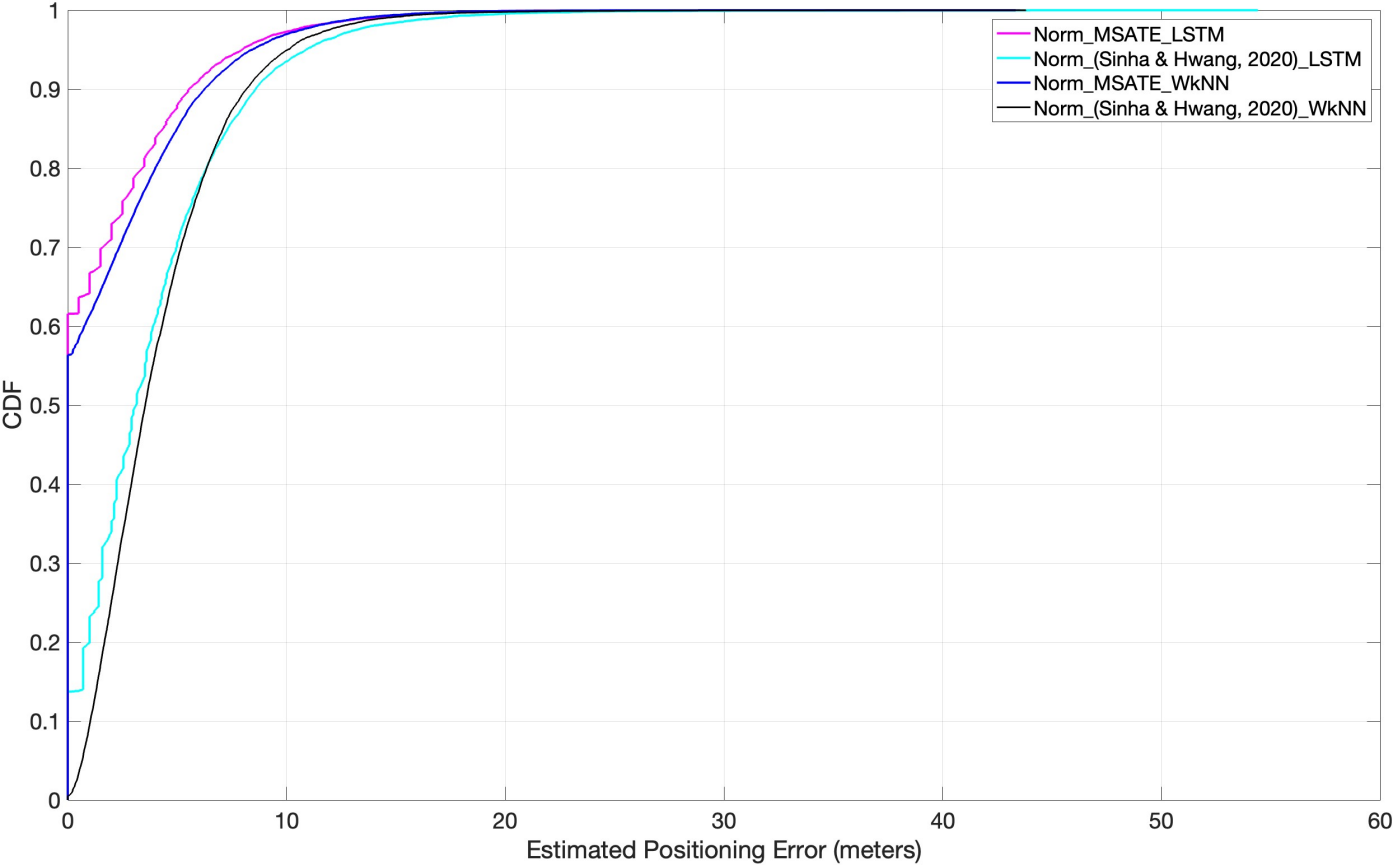
Comparison of cumulative positioning error probability (SMATE *vs* Sinha & Hwang, 2020) with both WkNN and LSTM.

The success of each augmentation strategy, MSATE, and that of Sinha & Hwang (2020), with similarity-based techniques, WkNN, and DL techniques, LSTM, is shown in Fig. 10 and Table 3. The figure and the table show that, compared to Sinha & Hwang (2020) with both LSTM and WkNN, the SMATE substantially influenced an improved positioning accuracy. That is due to the fact that our augmented new data samples were based on real data because it was based on plus-minus 4dBm (Maghdid et al., 2016b).

The classification accuracy on the test RSSI records was mentioned in Table 3 for each applied matching algorithm with different conditions. The proposed augmentation improved the positioning error by 33.1%; when MSATE was compared with the Sinha & Hwang (2020) augmentation algorithm. Further, if the proposed approach was applied with both normalization and augmentation, the positioning accuracy improved by 57.5%, when the proposed approach was compared with the Norm_ (Sinha & Hwang, 2020). To further understand the improvement in the positioning accuracy, Eq. (11) was calculated for both normalization and augmentation approaches.

(11) \begin{eqnarray*}ac{c}_{imp}= \frac{A-M}{A} \mathrm{ \ast }100\end{eqnarray*}

where *acc*_*imp*_ is positioning accuracy improvement in percentage; A represents the obtained positioning average errors in meters where the augmentation technique in Sinha & Hwang (2020) is adopted; and M is the average error when MSATE_LSTM is adopted. The same equation was applied to observe the improved positioning accuracy in percentages in those augmentations with normalization cases.

## Conclusions

The study reveals that simulators can be used to build and implement sane as genuine environments to avoid wasting time and effort during environmental surveys to generate the fingerprint dataset. In this study, OMNeT++ was used to design complicated real-world architecture for collecting RSSI vectors. Because fingerprint-based IP systems suffer from an unstable matching process, numerous well-known matching algorithms, including WkNN and LSTM, have been proposed. This research proposes a new augmentation technique (MSATE) to address the shortcomings of RSSI-based fingerprinting based on well-known matching techniques. Numerous experiments were performed to demonstrate how the suggested MSATE works as a database expansion to increase the efficiency of WkNN and LSTM. According to the results, the suggested augmentation and normalization enable the matching algorithms to conduct the localization in an impressively effective manner and improve positioning accuracy by 33.1% and 57.5%, respectively.

The main limitation of this study is the positioning accuracy as an error range of approximately 2 m is not enough for most indoor LBS applications. Additional studies are needed to evaluate the proposed approach when the number of WAPs is smaller or their signals do not exist in the vicinity. To this end, the proposed approach could be enhanced *via* integrating other existing indoor positioning technologies such as LoRa, LTE, or 5G cellular technologies.

##  Supplemental Information

10.7717/peerj-cs.1406/supp-1 Supplemental Information 1 The Original, Augmented, and Normalized datasets The OMNeT++ simulator simulates the third floor of the Faculty of Engineering at Koya University. The floor is chosen and constructed as an experimental setting in the proposed solution. The simulated area is approximately 56 ×77.6 square meters. In addition, four corridors, sixteen study halls, four computer laboratories, nine staff offices, three restrooms, and a large 13 m ×19 m Meeting Hall are included in the survey positions.
The entire region of the 3rd floor is considered for data collection. The whole area is divided into grids of 1m × 1m, resulting in dividing the region into 2,214 points, as illustrated in Fig. 6. At each sample point, RSSI data is recorded for one second. As a result, 22,140 records are collected.

10.7717/peerj-cs.1406/supp-2 Supplemental Information 2 Computer source code of the proposed Solution MSATE_Norm_LSTM and MSATE_Norm_WkNN The source code to implement the proposed solutions. This includes the following functions and snippets of codes:
(A) Augmentations function to perform the proposed MSATE (Augmentation) mechanism. MSATE is proposed to enhance the quantity of RSSI samples for each class or RP. In data analysis, one of the methods for increasing the quantity of RSSI data samples is known as “data augmentation.” When the data augmentation involves the creation of freshly produced synthetic RSSI data that is based on previously gathered datasets or the addition of slightly modified copies of RSSI data that already exists. It also serves as a regularizer and aids in reducing overfitting while training a Deep Learning model.
(B) Normalization function to normalize the RSSI Values. Normalization aims to maintain the RSSI values’ ranges while converting the values of a dataset’s numeric RSSI columns to a comparable scale.
(C) The MSATE_Norm_LSTM.m and MSATE_Norm_WkNN.m include the implementation codes regarding both proposed solutions, MSATE_Norm_LSTM and MSATE_Norm_WkNN respectively.

## Additional Information and Declarations

### Data Availability

The following information was supplied regarding data availability: The raw data and code are available in the Supplemental Files.
